# Regulatory and Operational Complexities of Conducting a Clinical Treatment Trial During an Ebola Virus Disease Epidemic

**DOI:** 10.1093/cid/cix1061

**Published:** 2017-12-01

**Authors:** Amanda M Rojek, Jake Dunning, Aleksandra Leliogdowicz, Lyndsey Castle, Mary Van Lieshout, Gail Carson, Foday Sahr, Piero Olliaro, Peter W Horby

**Affiliations:** 1 Epidemic Diseases Research Group Oxford (ERGO), Centre for Tropical Medicine and Global Health, University of Oxford, United Kingdom; 2 International Severe Acute Respiratory and Emerging Infection Consortium, Nuffield Department of Medicine, University of Oxford, United Kingdom; 3 Interdepartmental Division of Critical Care Medicine, Department of Medicine, University of Toronto, Ontario, Canada; 4 GOAL Global, Dun Laoghaire, Ireland; 5 Military 34 Hospital, Republic of Sierra Leone Armed Forces and College of Medicine and Allied Health Sciences, Freetown; 6 Centre for Tropical Medicine and Global Health, Nuffield Department of Medicine, University of Oxford, United Kingdom; 7 Special Programme for Research and Training in Tropical Diseases (TDR), World Health Organization, Geneva, Switzerland

**Keywords:** Ebola, epidemic, pandemic, clinical trial, viral hemorrhagic fever

## Abstract

The first phase II and III clinical trials for Ebola virus disease treatments were conducted during the West Africa outbreak. We report the operational practicalities of conducting a phase II clinical trial of TKM-130803 to international standards during this outbreak.

**(See the Viewpoints by Ellenberg et al on pages 1467–69.)**

Ebola virus disease (EVD)–specific treatments were proposed as a method to improve patient survival and curb further escalation of the West Africa (2013–2016) epidemic. The clinical trials undertaken to assess the most promising agents were unprecedented.

Subsequent reports lauded the trials as “ground-breaking” [1], but also criticized the paucity of definitive findings and the continued lack of licensed therapies [1, 2]. They advise that improving the ability to conduct analogous trials rapidly is a high priority in preparing for future epidemics [1, 3]. However, there are few records describing the practicalities of how this research was undertaken within the context of the outbreak [4] and the specific barriers to a swifter research response. Here, we report details for one such clinical trial.

## METHODS

The Rapid Assessment of Potential Interventions and Drugs for Ebola (RAPIDE) platform assessed World Health Organization–shortlisted experimental treatments during the epidemic. Two RAPIDE trials enrolled patients [5, 6], including the trial discussed here—a phase II clinical trial of the small interfering RNA lipid nanoparticle product TKM-130803 conducted in the GOAL Global Ebola Treatment Centre (ETC) in Sierra Leone. The World Health Organization prioritized this product for investigation on the basis of promising animal study data [7]. The TKM-230803 trial assessed safety and efficacy in patients with laboratory-confirmed EVD, with a primary outcome of day 14 survival, compared with historical controls. Although the full results of the trial are reported elsewhere [5], the trial closed to enrollment after reaching a prespecified futility boundary. For the current article, additional trial records and documents are presented Supplementary Methods [S1].

## RESULTS

### Trial Timeline

**Figure 1.**
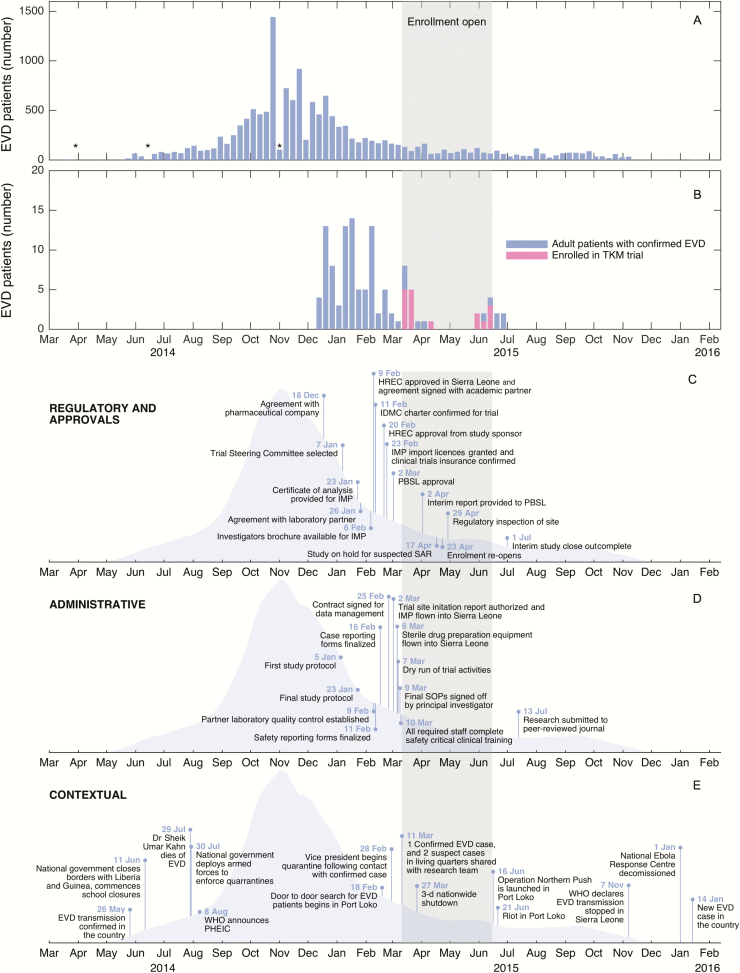
Timeline of RAPIDE TKM-130803 trial. *A*, Epidemiological curve for Sierra Leone, constructed using publicly available information from the World Health Organization (WHO) [8]; data are aggregated per calendar week. Asterisks denote underestimation due to correction in total case count by WHO. *B*, Timeline of admissions for adult patients with laboratory-confirmed Ebola virus disease (EVD) admitted to the GOAL Ebola Treatment Centre (unpublished data); data are aggregated per calendar week. *C*, Milestones to trial clearances and agreements. *D*, TKM-130803 administrative milestones. *E*, External events affecting Port Loko operating site; data are sourced from the United Nations [9], WHO [10], and personal records. Abbreviations: HREC, Human Research Ethics Committee; IDMC, Independent Data Monitoring Committee; IMP, Investigational Medicine Product; PBSL, Pharmacy Board of Sierra Leone; PHEIC, Public Health Emergency of International Concern; SOPs, standard operating procedure.

Figure 1 describes the TKM-130803 trial timeline, within the context of the outbreak. The TKM-130803 trial was the second trial to be prioritized within the platform, after a trial of brincidofovir that opened on 1 January 2015. Most TKM-130803 trial activities began after this time, and the trial began on 11 March 2015. The longest delays to starting the trial were caused by reaching research agreements with partners (39 days) and due mostly to concerns about legal liabilities. These agreements, and the time taken to formalize the research protocol (18 days), were most likely to affect possible enrollment of patients (though research protocol drafting was a faster process, it took place earlier during the outbreak, when the disease was spreading more rapidly) (detailed information on delays provided in the Supplementary Materials [S2]).

### Examples of Operational Complexity

#### Patient Enrollment

There was a 48-hour window for consent and enrollment, to maximize the potential efficacy of the antiviral and to prevent inadvertent enrollment of patients with convalescent phase EVD. Typically, 4 persons were required to enter the ETC’s high-risk zone (HRZ; where patients with suspected or confirmed EVD were located) to gain consent: a pair of English- and Krio-speaking trial clinicians, an interpreter fluent in local languages, and an independent witness. 

All participants in the trial were illiterate and so required independent verification of consent. For 2 participants, proxy consent was required because they were too unwell to be competent for decision making. Only written consent was considered appropriate for proxy consent, which created an unintended obstacle because the next of kin could be quarantined (n = 1) or also in an ETC (n = 1). If this relation could not be accessed by the trial team, he or she offered verbal assent, and then the next appropriate relation was approached for written consent. One patient became ineligible for enrollment owing to difficulty finding an appropriate next of kin, followed by extended travel time to meet the research team.

#### Patient Monitoring

TKM-130803 was provided as a 2-hour infusion via peripheral intravenous line, once daily for 7 days. Patients were monitored continuously during each infusion, and then a minimum of 5 regular observations were performed for an 8-hour period. All clinical work relevant to the trial was undertaken by trial- employed clinicians, but ETC clinicians remained responsible for overall patient care. Where possible, trial procedures were combined with routine care requirements to prevent duplication, although some procedures (eg, phlebotomy for TKM-130803 pharmacokinetics) remained trial specific.

The implication of this monitoring regimen was that significant human resources were required, especially because clinical care occurred in the HRZ where personal protective equipment (PPE) had to be worn. To prevent heat exhaustion, team members were restricted to entries lasting no more than 45–60 minutes, up to a maximum of 3 times per day (depending on temperature and humidity). To maintain safety, all staff worked in pairs. 

An example of the scheduling for a single patient is displayed in the Supplementary Results (S3). In total, a research team member entered the HRZ for patient-related activities at least 592 times during the trial. The actual number of entries is probably much higher, but we did not permanently record additional entries for training purposes, administrative duties, or unscheduled patient care (eg, for venous cannula replacement). There were no known PPE breaches, instances of heat strain or heat exhaustion, percutaneous sharps injuries, or “man-down” episodes involving trial staff. On 2 occasions, team members requested cancellation of a planned entry because they felt unwell.

#### Collecting Patient Data

Clinical data were recorded in writing on paper forms at the bedside. Writing while wearing PPE was difficult, so these forms were simplified and used large fonts. Clinical data were entered by one staff member and checked by another to minimize error in the challenging conditions within the HRZ.

Although the original source data would normally be filed for trial monitoring and regulatory inspections, infection prevention and control requirements prevented their transport out of the HRZ. Verbal handover of clinical information was not entertained owing to the potential for inaccuracies in conveying information. Flat-bed scanning of paper documents (with wireless and cable connections between the HRZ and the low-risk zone (LRZ; air-conditioned administrative area where PPE is not required)) was trialed but found to be too slow, with technical faults frequently encountered owing to heat, humidity, and possible environmental chlorine exposure. Instead, documents were placed on a post in the HRZ and photographed with a simple digital camera in the LRZ (illustrated in the Supplementary Results [S4]). 

Trial staff in the LRZ entered information from printed copies of the photographs onto an electronic case report form (Infermed MACRO; Elsevier). To enable real-time data reporting to the data safety monitoring board and reduce the risk of losing data because of hardware failure, the electronic case report forms were synchronized with the trial’s secure server (located in the United Kingdom) at least daily. Additional details of the strategies used for other on-site practices, including TKM130803 management, community engagement, and trial staff safety management, are provided in the Supplementary Results (S5).

## DISCUSSION

The TKM-130803 trial was operationally successful in that it was completed to a predefined statistical threshold, in a manner compliant with international regulatory frameworks for research and while maintaining the safety of the research team. The practical perspective of how this was achieved is relevant to the ongoing evaluations of science conducted during the epidemic, especially when these are meant to inform preparedness efforts.

Although this trial and others conducted during the outbreak were successful in fast-tracking to recruitment compared with trials conducted in other settings [11], the new target recommended by the Academy of Medical Sciences—that clinical trials are launched before the epidemic peak [1]—remains infeasible. Although a variety of solutions have been proposed [12, 13], our data suggest that preapproval of partnership agreements and predesigned protocols will be especially helpful.

Practical difficulties, as opposed to scientific or trial design issues, are a major contributor to delayed and inadequate recruitment for clinical trials [14]. The additional operational complexities during outbreaks can be significant may be underappreciated. Key constraints for EVD include stringent infection prevention and control requirements impairing ready access to patients and the expanded breadth of logistical activities undertaken by research staff working in a resource limited environment. The consequences can include a difficulty in scaling up recruitment (or initiating multicenter recruitment) without threatening staff or patient safety or detracting from the immediate humanitarian priorities of the clinical team.

The most significant improvement to the operational feasibility of outbreak clinical research will occur when research is integrated into the overall outbreak response. When high-quality clinical data are captured according to standard operating procedures and using agreed-on data standards, the process meets the shared needs of clinical and research teams and minimizes duplication. The benefits are multifold; patients will be protected from additional examinations or interventions, the utility of clinicians is maximized, and multisite research will be simpler to conduct. Technological and human factors advancements that will automate or simplify the collection of clinical data have particular potential for infectious diseases that require PPE to be worn.

## Supplementary Data

Supplementary materials are available at *Clinical Infectious Diseases* online. Consisting of data provided by the authors to benefit the reader, the posted materials are not copyedited and are the sole responsibility of the authors, so questions or comments should be addressed to the corresponding author.

Supplementary Materials

## Notes

***Acknowledgments.*** We thank trial participants and the following groups for their collaboration: GOAL Global, Public Health England, the Department for International Development, and staff at the participating sites. The following were instrumental to the operational management of the TKM-130803 trial: Rugiatu Conteh and Margarite D’Amelio (GOAL Global); Catrin Moore (University of Oxford); Merete Storgaard (Aarhus University); and Vanessa Vy and Amidu Dumbaya (London School of Hygiene and Tropical Medicine). Graeme Macgilchrist (University of Oxford) assisted with manuscript preparation.

***Disclaimer.*** The funders had no role in study design, data collection and analysis, decision to publish, or preparation of the manuscript.

***Financial support.*** This work was supported by the Wellcome Trust of Great Britain (grant 106491/ Z/14/Z and 089275/Z/09/Z) and by the EU FP7 project PREPARE (grant 602525). A. M. R. is funded by a Rhodes Scholarship.

***Potential conflicts of interest.*** All authors: No reported conflicts. All authors have submitted the ICMJE Form for Disclosure of Potential Conflicts of Interest. Conflicts that the editors consider relevant to the content of the manuscript have been disclosed.
