# Supplementary material for: Regulatory and Operational Complexities of Conducting a Clinical Treatment Trial During an Ebola Virus Disease Epidemic
Source: Clin Infect Dis. 2017 Dec 1;66(9):1454–7. doi: 10.1093/cid/cix1061 (PMC5905621; doi:10.1093/cid/cix1061)
Supplement: Supplementary Materials [file cix1061_suppl_supplementary_materials.docx]

**Supplementary materials**

**S1: Methods**

**Data sources:** For this work, the investigator site file, internal team communications and reports, project management records, regulatory records, and meeting minutes were reviewed by the primary author (AR) to aid recollection of the sequence of events. Individual investigators were contacted where additional verification was required. Epidemiological information was extracted from publicly available WHO data, and admission records at the research site were provided by the clinical care partner, GOAL Global.

**Data interpretation:** Where study timelines are produced, the term `delay' refers to the time period between initiating and completing an activity. All reporting of `days' refers to the number of calendar days. Where we report the number of potential patients that could be enrolled during a period, this is estimated based on the proxy indicator of adult EVD admissions to the GOAL ETC during that period, as these represent patients who would be screened for eligibility.

**S2: Details of regulatory or administrative milestones.**

| **Regulatory or administrative milestone** | **Time taken** | | | **New EVD cases during delay period (n)** | |
| --- | --- | --- | --- | --- | --- |
|  | Start date  (dd/mm) | Final date  (dd/mm) | Duration  (days) | Sierra Leone^ | GOAL ETC (Adults) |
| **Research agreements with partners** | | | | | |
| Laboratory (Public Health England) | 26/01/2015 | 06/03/2015 | 39 | 948-1279 | 28 |
| Academic (College of Medicine and Allied Health Sciences) | 26/01/2015 | 19/02/2015 | 24 | 585-961 | 23 |
| Site (GOAL Global) | 26/01/2015 | 06/03/2015 | 39 | 948-1279 | 28 |
| Pharmaceutical (Tekmira Pharmaceuticals) |  | 18/12/2014 | N/A | N/A |  |
| **Human Research Ethics Committee (HREC) Approvals** | | | | | |
| Prepare submission (Sierra Leone HREC) | 23/01/2015 | 27/01/2015 | 4 | <178 | 3 |
| Review process (Sierra Leone HREC) | 27/01/2015 | 10/02/2015 | 14 | 222-594 | 19 |
| Prepare submission (trial sponsor HREC) | 23/01/2015 | 29/01/2015 | 6 | <400 | 6 |
| Review process (trial sponsor HREC) | 29/01/2015 | 20/02/2015 | 22 | 363-783 | 23 |
| Prepare submission (WHO for opinion) |  | 20/02/2015 | N/A | N/A |  |
| **Protocol development** | | | | | |
| Drafting | 05/01/2015 | 23/01/2015 | 18 | 560-1072 | 28 |
| **IMP management** | | | | | |
| Drug release | 19/02/2015 | 09/03/2015 | 18 | 165-516 | 5 |
| Import license | 16/02/2015 | 23/02/2015 | 7 | <367 | 5 |
| Shipment of IMP | 27/02/2015 | 02/03/2015 | 3 | <165 | 3 |
| **Data Management** | | | | | |
| Database design, set-up and testing | 23/02/2015 | 16/03/2015 | 21 | 318-648 | 10 |
| Case reporting form development | 05/02/2015 | 10/03/2015 | 33 | 532-879 | 14 |
| **Other Required Documents** | | | | | |
| Study insurance | 03/02/2015 | 23/02/2015 | 20 | 363-783 | 12 |
| European Medicines Agency opinion | 06/02/2015 | 10/02/2015 | 4 | <194 | 13 |
| Table S2. Time taken to meet key regulatory requirements for clinical trial, and the implications of these delays on possible enrolment. ^ figures according to WHO weekly report. | | | | | |

**S3: Daily operations of RAPIDE TKM-130803 trial.**

**
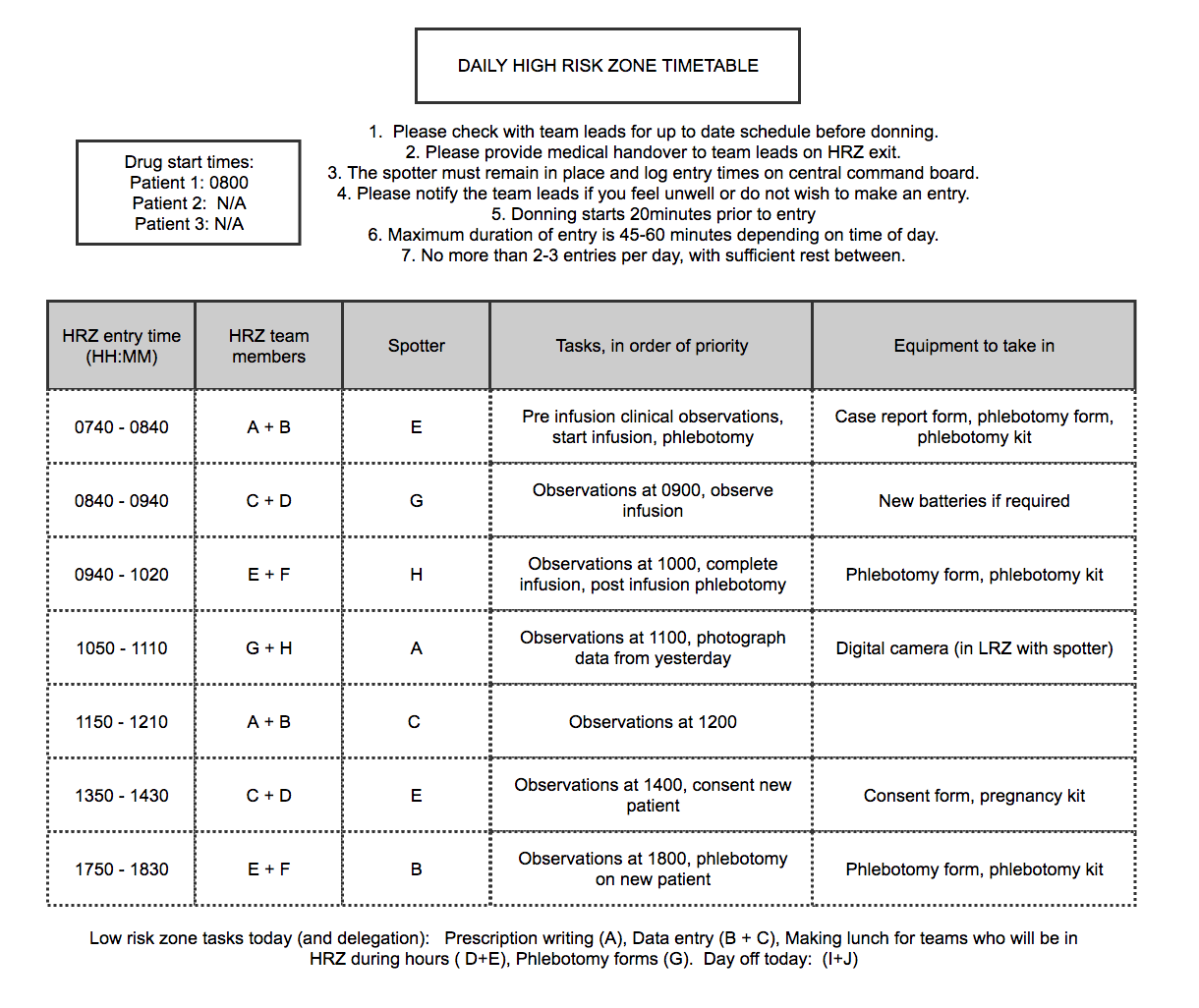
**

Figure S3: Example of the daily run-sheet housed in the LRZ (low-risk zone), demonstrating the human resources required to safely monitor one patient receiving TKM-130803 per day. A minimum of 10 clinical staff were required to monitor one patient through their treatment course. 592 individual team member entries were required to monitor all patients in the trial. The term *clinical observations* refers to assessment of pulse rate, blood pressure, respiratory rate, temperature, level of consciousness, signs and symptoms of EVD, SARS and SUSARS.

**S4: Transfer of data from HRZ to LRZ.**

Figure S4: Data transfer options from the HRZ.

**S5: Additional operational considerations**

*Management of TKM-130803*

Due to limited commercial flight availability TKM-130803 was transported in a temperature controlled hold on a routine United Kingdom Royal Air Force flight. Initially, only 14 treatment courses were shipped in case there was a disruption to chain of custody or cold-chain. The temperature sensitive drug was packed in shipment boxes that contained electronic temperature monitors (TempTale® 4 USB, Sensitech Inc, USA).

Once on-site, TKM-130803was stored in a temperature-controlled, locked vaccine refrigerator with an automatic switchover to a back-up generator in the event of power failure. Temperature was monitored daily.

TKM-130803was supplied as an aqueous dispersion that required dilution before administration. However, as there were no aseptic drug preparation facilities available, trial pharmacists were deployed and trained in use of a portable positive pressure aseptic preparation unit designed for field use (The Posi-Dome™ Basic, Banthrax Corporation, Dayton, Ohio, USA).

Community engagement.

Specific community engagement concerns in Port Loko included recent attacks on ambulances and burial teams[1] and perceptions of the outbreak as a supernatural event[2].

A multifaceted public engagement approach was led by the trial principal investigator (an experienced senior clinician from Sierra Leone). The trial was presented to local chiefs and community leaders at an Ebola task force meeting and some of these representatives visited the research group subsequently. Affected communities received information about the trial through the existing community engagement infrastructure of GOAL Global. ETC staff, some of whom were members of affected communities attended Krio and English language question and answer sessions and were provided with plain language summaries of the trial and the research team promoted an ‘open door’ policy for staff who had further questions. Other organisations involved in the regional EVD response received briefings through the District Ebola Response Committee meetings.

Staff safety.

The strategy used to prevent infection of trial staff included PPE training and continuous monitoring of PPE use; a strict ‘no touch’ and social distancing policy; health surveillance, including the immediate reporting of symptoms that could represent EVD, and body temperature monitoring when needed. A senior physician was responsible for the health and safety of team members on site. They were supported from the UK by a 24-hour on call rota that included a team coordinator and a duty doctor (both senior physicians), and an experienced health and safety officer.

Standard operating procedures were developed for heat exposure, snake or scorpion envenomation, motor vehicle collisions, a deteriorating security situation (such as civil unrest), potential EVD exposure (e.g. needle-stick injury), and medical illness (particularly management of illness that could represent EVD). Several of these protocols were utilised to manage incidents. For psychological support RAPIDE field staff received pre- and post-departure briefings, access to an independent counselling service if needed, as well as a formal group debrief.

**References**

1. Wilkinson A, Fairhead J. Comparison of social resistance to Ebola response in Sierra Leone and Guinea suggests explanations lie in political configurations not culture. Crit Public Health. 2017;27(1):14-27.

2. World Health Organization. When Ebola came calling: How communities in Sierra Leone faced the challenge 2014. Available from: <http://www.who.int/features/2014/ebola-community-care/en/> (cited May 3, 2017).
